# Comparisons of perioperative and long-term outcomes of laparoscopic versus open gastrectomy for advanced gastric cancer after neoadjuvant therapy: an updated pooled analysis of eighteen studies

**DOI:** 10.1186/s40001-023-01197-1

**Published:** 2023-07-05

**Authors:** Hua-Yang Pang, Xiu-Feng Chen, Li-Hui Chen, Meng-Hua Yan, Zhi-Xiong Chen, Hao Sun

**Affiliations:** 1 Gastrointestinal Cancer Center, Chongqing University Cancer Hospital, Chongqing, China; 2 Chongqing Key Laboratory of Translational Research for Cancer Metastasis and Individualized Treatment, Chongqing University Cancer Hospital, Chongqing, China

**Keywords:** Gastric cancer, Neoadjuvant therapy, Laparoscopic gastrectomy, Open gastrectomy, Meta-analysis

## Abstract

**Background:**

Outcomes of laparoscopic surgery in advanced gastric cancer patients who received neoadjuvant therapy represent a controversial issue. We performed an updated meta-analysis to evaluate the perioperative and long-term survival outcomes of laparoscopic gastrectomy (LG) versus conventional open gastrectomy (OG) in this subset of patients.

**Methods:**

Electronic databases including PubMed, Embase, Web of Science, the Cochrane Central Register of Controlled Trials and China National Knowledge Infrastructure were comprehensively searched up to May 2023. The short-term and long-term outcomes of LG versus OG in advanced gastric cancer patients undergoing neoadjuvant therapy were evaluated. Effect sizes with 95% confidence intervals were always assessed using random-effects model. The prospective protocol was registered with PROSPERO (CRD42022359126).

**Results:**

Eighteen studies (2 randomized controlled trials and 16 cohort studies) involving 2096 patients were included. In total, 933 patients were treated with LG and 1163 patients were treated with OG. In perioperative outcomes, LG was associated with less estimated blood loss (MD = − 65.15; *P* < 0.0001), faster time to flatus (MD = − 0.56; *P* < 0.0001) and liquid intake (MD = − 0.42; *P* = 0.02), reduced hospital stay (MD = − 2.26; *P* < 0.0001), lower overall complication rate (OR = 0.70; *P* = 0.002) and lower minor complication rate (OR = 0.69; *P* = 0.006), while longer operative time (MD = 25.98; *P* < 0.0001). There were no significant differences between the two groups in terms of proximal margin, distal margin, R1/R2 resection rate, retrieved lymph nodes, time to remove gastric tube and drainage tube, major complications and other specific complications. In survival outcomes, LG and OG were not significantly different in overall survival, disease-free survival and recurrence-free survival.

**Conclusion:**

LG can be a safe and feasible technique for the treatment of advanced gastric cancer patients receiving neoadjuvant therapy. However, more high-quality randomized controlled trials are still needed to further validate the results of our study.

**Supplementary Information:**

The online version contains supplementary material available at 10.1186/s40001-023-01197-1.

## Background

Gastric cancer (GC) is the third leading cause of cancer-related deaths worldwide [1, 2]. For patients with locally advanced gastric cancer (AGC), radical gastrectomy combined with D2 lymphadenectomy has been regarded as the only promising technique for curing the disease in both eastern and western countries [3]. In addition, the implementation of perioperative multi-mode therapy can also improve the oncological outcomes of patients [4]. Currently, several large-scale clinical trials such as MAGIC [5], FLOT4 [6], RESONANCE [7] and RESOLVE [8] have confirmed that neoadjuvant therapy (NAT) combined with surgical resection can improve the chances of R0 resection, eliminating possible micro-metastases and improve long-term survival relative to upfront surgery [9, 10]. The National Comprehensive Cancer Network (NCCN) recommends that NAT should be administered to all AGC patients, and it has been included in the standardized multi-mode treatment of AGC in many countries around the world [11].

Since Kitano et al. [12] first reported the application of laparoscopic technique for distal gastrectomy (LG) in 1994, LG has emerged as a standard surgical approach for the treatment of early GC due to the comparable surgical and long-term results relative to open gastrectomy (OG). In recent years, three large-scale randomized controlled trials (RCTs) from JLSSG0901 [13], KLASS-02 [14] and CLASS-01 [15] have further extended the indications of LG to AGC. However, in the context of NAT, therapy-induced tissue edema and fibrosis, vascular fragility, and normal anatomic disappearance pose new challenges to the application of laparoscopic technique in those patients [16, 17]. Laparoscopic technique has the advantages of a magnified surgical field and good maneuverability. A previous meta-analysis by Liao et al. [18] demonstrated that patients in the LG group had a quasi-significantly less complication rate and faster time to flatus than patients in the OG group, while other clinical outcomes were not significantly different between the two groups. Nevertheless, this meta-analysis included 6 studies with only 704 patients available, making many of the findings less statistically powerful. Therefore, it remains uncertain whether laparoscopic techniques can be used as an alternative to open surgery in AGC patients receiving NAT.

In the last 2 years, a growing body of additional research has been addressed to further explore the application value of LG in neoadjuvant gastric cancer patients. Therefore, based on existing evidence, we performed an updated meta-analysis to investigate the perioperative and survival outcomes of LG relative to OG in AGC patients following NAT.

## Methods

Our meta-analysis was accomplished in line with the requirements from PRISMA statement, to identify studies comparing the perioperative and long-term outcomes of LG vs. OG for AGC patients who underwent NAT. The prospective protocol was registered with PROSPERO (CRD42022359126).

### Search strategy

Electronic datasets including PubMed, Embase, Web of Science, the Cochrane Central Register of Controlled Trials and China National Knowledge Infrastructure were systematically examined for relevant studies until to May 31, 2023. The following search strategy was used to perform the study retrieval: (“open” [Title/Abstract] OR “laparotomy” [Title/Abstract]) AND (“laparoscopic” [Title/Abstract] OR “laparoscopy” [Title/Abstract]) AND (“neoadjuvant” [Title/Abstract] OR “preoperative” [Title/Abstract] OR “perioperative” [Title/Abstract]) AND (“gastric cancer” [Title/Abstract] OR “stomach cancer” [Title/Abstract] OR “stomach neoplasm” [Title/Abstract]). During the search process, language restrictions were not applied. Moreover, references to previously published reviews and included studies were also manually searched for additional reports. The literature search was conducted by two investigators independently (HY-P and XF-C).

### Inclusion and exclusion criteria

The inclusion criteria were formulated through the PICOS approach [19] as follows. **P:** AGC patients who underwent NAT; **I:** laparoscopic gastrectomy; **C:** open gastrectomy; **O:** perioperative and survival outcomes; **S:** comparative studies including RCTs, cohort studies and case-controlled studies.

The exclusion criteria were studies (1) reported as case reports, conferences, reviews, and abstracts; (2) with mixed cancers; (3) with overlapping data.

### Data extraction

A standardized EXCEL form was designed for data extraction. Two independent reviewers (HY-P and XF-C) performed this procedure and cross-checked all the results. Any discrepancies were resolved by a third reviewer (H S). The following data from each study were extracted and recorded: first author, publication year, study interval, country, study design and sample size, age, sex, body mass index (BMI), American Society of Anesthesiologists (ASA) classification, neoadjuvant regimen, tumor size, gastrectomy extent, follow-up time, perioperative outcomes and survival outcomes.

### Outcome of interest and definitions

Perioperative and long-term survival outcomes between the LG and OG groups were assessed in this study. Perioperative outcomes included (1) intraoperative and postoperative recovery outcomes: operative time, estimated blood loss, proximal margin, distal margin, R1/R2 rate, number of retrieved lymph nodes, time to first flatus, time to first liquid intake, time to remove gastric tube, time to remove drainage tube, and postoperative hospital stay; and (2) postoperative complications which occurred during hospitalization or within 30 days after gastrectomy were defined and graded by Clavien-Dindo (CD) classification system [20], including total complications (CD I-V), minor complications (CD I-II), major complications (CD III-V), anastomotic leakage, pancreatic complications, intra-abdominal hemorrhage, intra-abdominal abscess, surgical site infection, lymphatic leakage, pulmonary infection and Ileus. Long-term survival outcomes included overall survival (OS), disease-free survival (DFS) and recurrence-free survival (RFS).

### Quality assessment

In terms of the literature quality of included studies, the Cochrane Risk-of-Bias 2.0 tool (ROB 2.0) [21] were utilized to evaluate the risk of bias for included RCTs, which consists of five domains: randomization process, deviations from intended intervention, missing data, outcome measurement and selection of reported result. While for cohort studies, the Risk of Bias in Non-Randomized Studies-of Interventions tool (ROBINS-I) [22] was applied, which consists of seven domains: confounding factors, selection of participants, classification of interventions, deviation from intended interventions, missing data, outcome measurement and selection of the reported result.

### Statistical analysis

Dichotomous variables, continuous variables, and survival outcomes were analyzed with using the odds ratios (ORs), mean differences (MDs) and hazard ratios (HRs) with their 95% confidence intervals (CIs), respectively. For studies reporting continuous variables as median with range or inter-quartile range, we converted data into mean with standard deviation (SD) according to the approach described by McGrath et al. [23]. For studies that HRs with 95% CIs were not provided, we extracted data from survival curves and calculated them following the methods developed by Tierney et al. [24]. Heterogeneities of pooled outcomes were assessed using *I*^2^ statistic [25]. A random-effects model was always performed, due to common clinical and conceptual variance across included studies. In addition, meta-regression analysis and subgroup analysis were performed to investigate the source of heterogeneity and robustness of pooled results. Begg’s funnel plots were applied to test potential publication bias of pooled outcomes when there were at least ten studies included. The pooled results were considered statistically significant at two tailed *P* < 0.05. Review Manager Software, version 5.3 (Cochrane, London, UK) and Stata, version 12.0 (Statacorp, College Station, TX) were used to perform all statistical analyses.

## Results

### Study characteristics and risk of bias

**Fig. 1:**
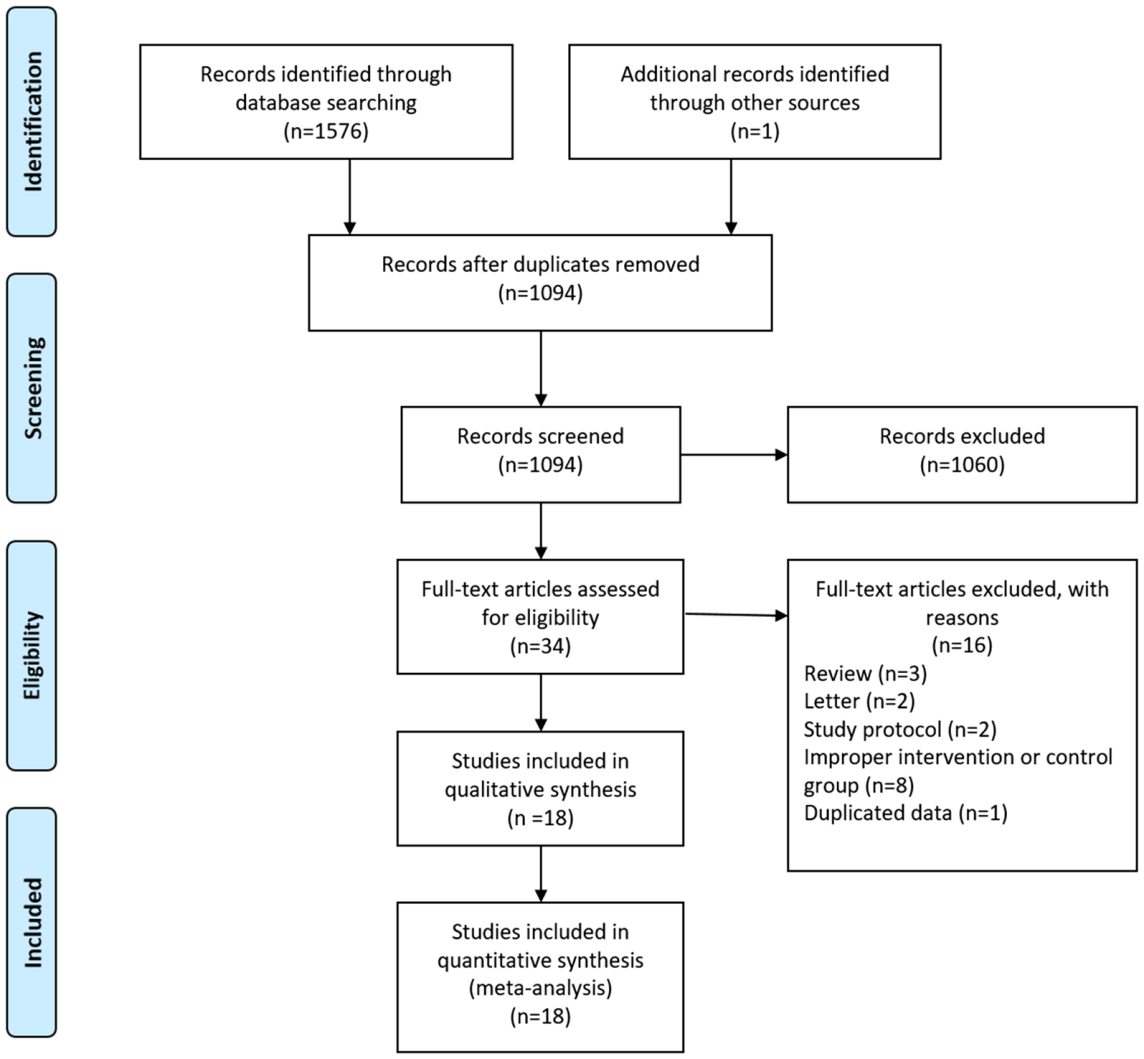
The PRISMA Flowchart of study selection

As shown in Fig. 1, a total of 1576 potential studies were yielded after systematically searching. Through careful title, abstract and full text assessment, 2 RCTs [26, 27] and 16 cohort studies [28–43] were ultimately enrolled in this study. As shown in Table 1, a total of 2096 patients (933 in the LG group and 1163 in the OG group) were included in the present study. These studies were published between 2014 and 2023, with a sample size ranging from 44 to 270. Of these studies, 15 originated in China and another 3 were from Japan, Egypt and Europe. The median follow-up time of included studies ranged from 31 to 69 months. For neoadjuvant therapy, most studies used chemotherapy as a preoperative treatment and a few studies [29, 31, 37, 42] also included targeted therapy and concurrent chemoradiation in the preoperative treatment strategy. As for the baseline characteristics including age, sex, ASA score, BMI, tumor size and gastrectomy extent between the two groups, 15 studies did not report statistically significant differences in these variables. However, 2 studies were not matched according to tumor size, where the tumor size in the LG group was smaller in the study by Ge et al. [30], but larger in the study by Li et al. [35]. Besides, one study by Wu et al. [40] showed a difference in gastrectomy extent, with the total gastrectomy being larger on average in the OG group (Table 2).

**Table 1:** Basic information of included studies

References	Country	Study design	Study interval	Sample size (LG/OG)	Neoadjuvant regimen	Follow-up, months
Cui et al. 2022 [28]	China	R; S	2012–2019	136 (61/75)	SOX; XELOX; SF; DCF	69 (range, 1–112)
Fujisaki et al. 2020 [29]	Japan	R; S	2009–2018	49 (20/29)	SP; SOX; Trastuzumab + SOX; Trastuzumab + CAPOX	38 (range, 3–115)
Ge et al. 2022 [30]	China	R; S	2017–2019	153 (77/76)	XELOX; FOLFOX; SOX; FLOT	NA
Hu et al. 2022 [31]	China	R; S; PSM	2011–2018	138 (69/69)	SOX; XELOX; CS; FOLFOX; TP; ECF; DCF; DCX; Chemoradiotherapy with S-1	45 (range, 3–94)
Hu et al. 2022 [32]	China	R; S; PSM	2018–2020	66 (34/32)	SOX; FLOT	NA
Khaled et al. 2021 [34]	Egypt	R; M	NA	84 (41/43)	XELOX	NA
Li et al. 2016 [35]	China	P; S	2012–2014	44 (20/24)	SOX; CAPOX; FOLFOX7	NA
Li et al. 2019 [26]	China	RCT; S	2015–2017	95 (45/50)	XELOX	NA
Shen et al. 2020 [33]	China	R; S	2018–2020	90 (45/45)	XELOX	NA
van der Wielen et al. 2020 [27]	Europe	RCT; M	2015–2018	96 (47/49)	ECC; ECF; EOX; FOLFOX; FLOT	NA
Wang et al. 2014 [36]	China	R; S	2011–2014	120 (68/52)	XELOX	NA
Wang,2016 [37]	China	R; S	2013–2014	134 (67/67)	XELOX	NA
Wang et al. 2020 [38]	China	R; S	2007–2016	270 (49/221)	XELOX; FOLFOX; SOX; SP; TXT + XELOX; TCF; DOS; TXT + SP; Fluoropyrimidine-based chemoradiotherapy	NA
Wang et al. 2021 [39]	China	R; S; PSM	2013–2018	69 (23/46)	DC; DS; DX; EP; FOLFIRI; POS; S1; SEEOX; SOX; CAPOX	NA
Wu et al. 2022 [40]	China	R; S	2017–2020	154 (52/102)	SOX; XELOX; DCF; XP	31 (range, 2–60)
Xi et al. 2020 [41]	China	R; S; PSM	2013–2016	90 (45/45)	SOX; XELOX	OG:39 (range, 12–49) LG: 33 (range, 9–58)
Zheng et al. 2023[42]	China	R; S	2008–2018	146 (89/57)	SOX; XELOX; DS; FOLFOX4; Oxaliplatin + Apatinib	NA
Zhong et al. 2023[43]	China	R; S; PSM	2015–2019	162 (81/81)	SOX; FLOT; DOS; DCF	NA

LG: laparoscopic gastrectomy; OG: open gastrectomy; R: retrospective; P: prospective; RCT: randomized controlled trial; S: single center; M: multiple center; PSM: propensity score matching analysis; TG: total gastrectomy; PG: proximal gastrectomy; DG: distal gastrectomy; NA: not available

**Table 2:** Clinicopathologic features of included studies

References	Age, years		Sex (M/F)		ASA score (≥ 3)		BMI, kg/m^2^		Tumor size, cm		Gastrectomy extent
	LG	OG	LG	OG	LG	OG	LG	OG	LG	OG	
Cui et al. 2022 [28]	57.6 ± 10.4	56.8 ± 12.0	47/14	59/16	NA	NA	22.8 ± 2.7	23.7 ± 3.3	4.0(2.5–6.5)	4.0(2.0–6.0)	TG
Fujisaki et al. 2020 [29]	71.5(44.0–79.0)	67.0(46.0–80.0)	13/7	22/7	2	1	21.9 (17.0–26.5)	21.9 (14.8–29.5)	7.5 (3.0–15.0)	5.0 (2.0–10.0)	DG/TG
Ge et al. 2022 [30]	60.4 ± 9.4	59.3 ± 10.6	53/24	59/17	NA	NA	23.0 ± 3.2	23.7 ± 2.9	3.6 ± 1.7	4.5 ± 2.6	DG/TG
Hu et al. 2022 [31]	53.4 ± 13.4	53.9 ± 12.7	53/16	52/17	4	5	22.6 ± 3.1	22.8 ± 3.3	5.4 ± 3.3	5.4 ± 3.1	TG
Hu et al. 2022 [32]	NA	NA	18/16	15/17	2	1	NA	NA	NA	NA	TG
Khaled et al. 2021 [34]	62.3 ± 4.5	64.0 ± 10.7	20/21	20/23	NA	NA	NA	NA	NA	NA	DG/TG
Li et al. 2016 [35]	53.5 ± 9.2	56.0 ± 9.2	13/7	21/3	2	3	22.4 ± 3.7	23.0 ± 3.1	3.7 ± 1.9	2.5 ± 1.3	DG
Li et al. 2019 [26]	59.0(52.0–65.0)	61.0(55.0–64.0)	32/13	34/16	NA	NA	23.5 (20.9–25.0)	22.8 (21.3–24.7)	6.0 (5.1–7.9)	6.2 (5.5–7.5)	DG/TG
Shen et al. 2020 [33]	62.1 ± 2.2	62.7 ± 2.2	23/22	24/21	NA	NA	NA	NA	NA	NA	TG
van der Wielen et al. 2020 [27]	59.4 ± 12.5	61.8 ± 10	28/19	32/17	13	12	26.5 ± 4.8	25.2 ± 4.0	NA	NA	TG
Wang et al. 2014 [36]	52.9 ± 15.1	51. 6 ± 8. 2	39/29	31/21	NA	NA	NA	NA	NA	NA	TG/PG/DG
Wang et al. 2016 [37]	58.4 ± 12.5	58.9 ± 12.1	41/26	42/25	NA	NA	NA	NA	NA	NA	NA
Wang et al. 2020 [38]	54.4 ± 10.9	54.9 ± 11.3	34/15	154/67	NA	NA	NA	NA	NA	NA	TG/PG/DG
Wang et al. 2021 [39]	60.1 ± 9.7	59.7 ± 8.7	18/5	36/10	2	4	23.7 ± 3.8	23.3 ± 3.7	3.43 ± 1.30	3.41 ± 1.70	TG
Wu et al. 2022 [40]	59.6 ± 9.4	61.1 ± 8.7	38/14	18/84	NA	NA	21.8 ± 2.8	21.9 ± 2.9	NA	NA	TG/PG/DG
Xi et al. 2020 [41]	57.1 ± 6.6	59.6 ± 7.5	36/9	33/12	6	8	23.7 ± 3.0	23.9 ± 3.3	NA	NA	TG/PG/DG
Zheng et al. 2023 [42]	60.3 ± 10.1	63.3 ± 11.3	69/20	46/11	NA	NA	NA	NA	5.2 ± 3.3	6.1 ± 3.6	TG
Zhong et al. 2023 [43]	59.0 ± 8.8	60.0 ± 9.7	59/22	57/24	40	41	23.7 (22.4–25.0)	23.5 (21.0–25.7)	3.3 (2.0–5.25)	3.5 (2.0–5.0)	TG/PG/DG

LG: laparoscopic gastrectomy; OG: open gastrectomy; TG: total gastrectomy; PG: proximal gastrectomy; DG: distal gastrectomy; M: male; F: female; ASA: American Society of Anesthesiologists classification; BMI: body mass index; NA: not available

### Risk of bias

**Fig. 2:**
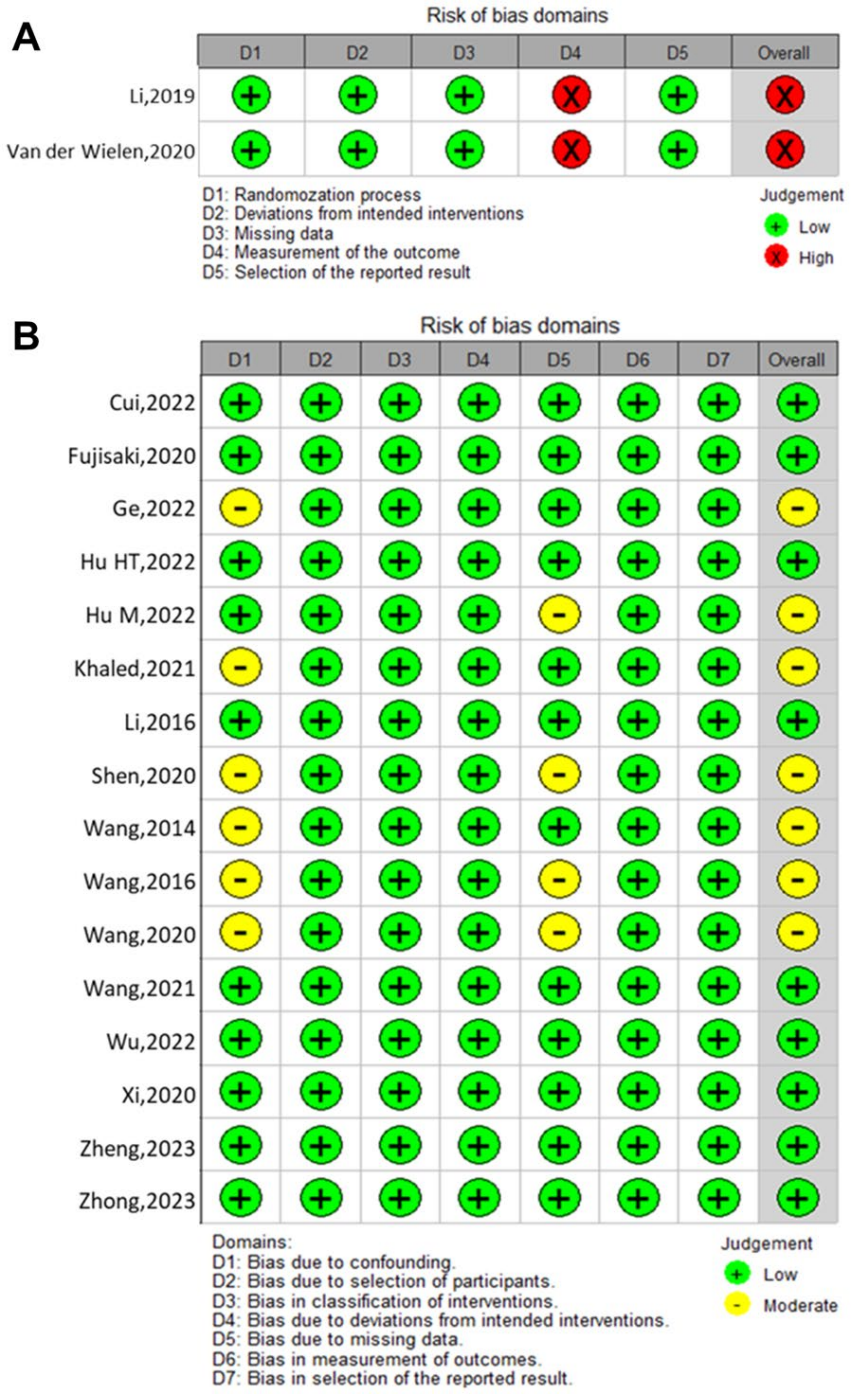
Bias risk summary of each element in the included randomized controlled trials (**A**) and cohort studies (**B**)

The 2 RCTs were evaluated using the ROB 2.0 tool, and both were deemed to be at high risk in the domain of the outcome measurement due to the surgical nature of these trials, which made it impossible to blind participants and investigators to the procedure (Fig. 2A). The 16 cohort studies were evaluated using the ROBINS-I tool, and 7 of them were moderate risk in the overall risk of bias due to 6 of them had a moderate risk in the domain of confounding factors and 4 of them had a moderate risk in the domain of missing data (Fig. 2B).

### Meta-analysis of outcomes

#### Intraoperative and recovery outcomes

**Fig. 3:**
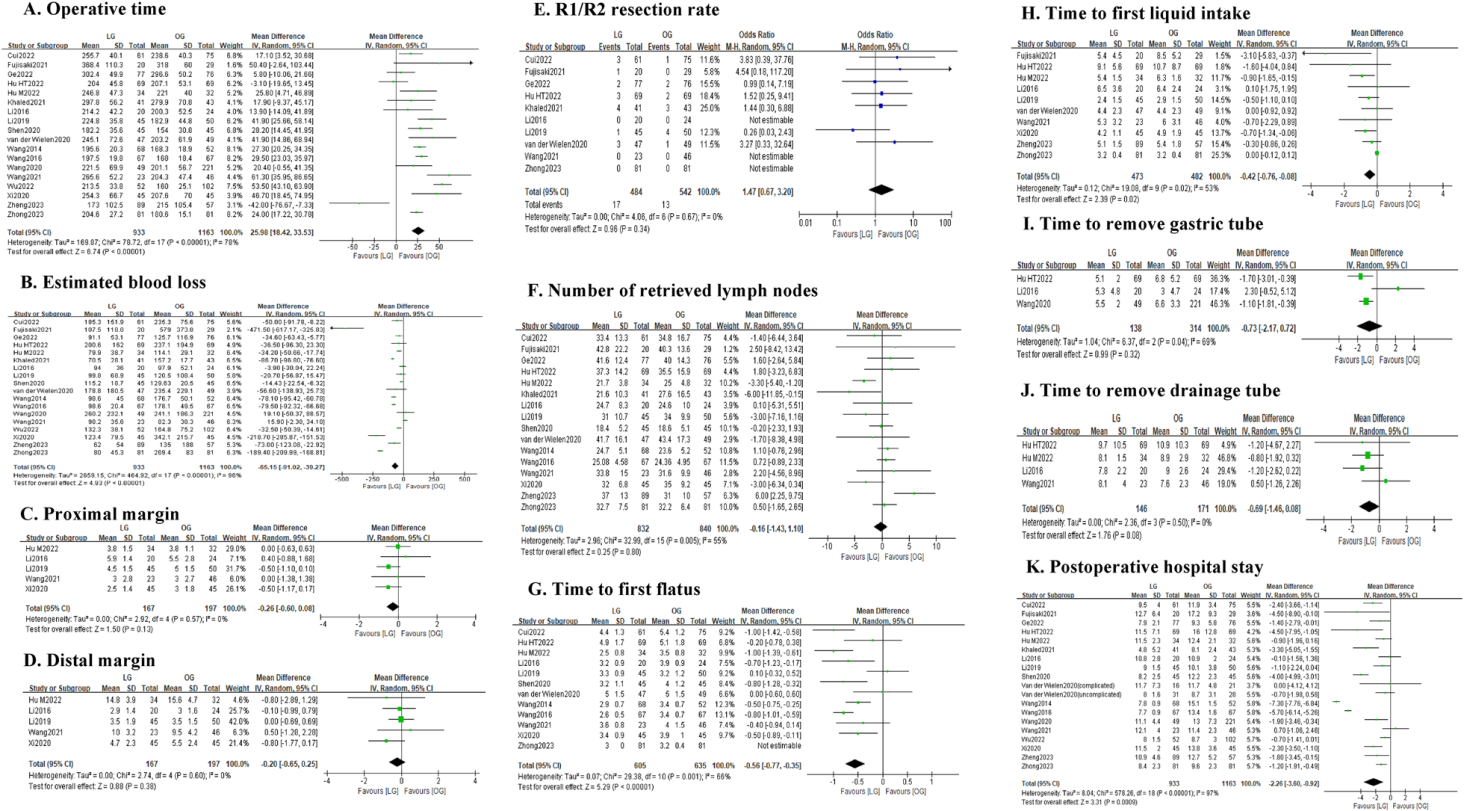
Forest plot assessing intraoperative and recovery outcomes between the LG and OG groups. **A** Operative time; **B** estimated blood loss; **C** proximal margin; **D** distal margin; **E** R1/R2 resection rate; **F** number of retrieved lymph nodes; G. time to first flatus; **H** time to first liquid intake; **I** time to remove gastric tube; **J** time to remove drainage tube and **K** postoperative hospital stay

As shown in Fig. 3 and Table 3, patients in the LG group had a significantly less estimated blood loss (MD = − 65.15; 95% CI − 91.02 to − 39.27; *P* < 0.0001; *I*^2^ = 96%) but longer operative time (MD = 25.98; 95% CI 18.42–33.53; *P* < 0.00001; *I*^2^ = 78%) than the OG group. There were no significant differences between the two groups in number of retrieved lymph nodes (MD = − 0.16; 95% CI − 1.43 to 1.10; *P* = 0.80; I^2^ = 55%), proximal margin (MD = − 0.20; 95% CI − 0.65 to 0.25; *P* = 0.38; *I*^2^ = 0%), distal margin (MD = − 0.26; 95% CI − 0.60 to 0.08; *P* = 0.13; *I*^2^ = 0%) and R1/R2 rate (OR = 1.47; 95% CI 0.67–3.20; *P* = 0.34; *I*^2^ = 0%). In postoperative recovery, patients in the LG group had a shorter length of hospital stay (MD = − 2.26; 95% CI -3.60 to − 0.92; *P* < 0.0001; *I*^2^ = 97%), time to first flatus (MD = − 0.56; 95% CI − 0.77 to − 0.35; *P* < 0.0001; *I*^2^ = 66%) and time to first liquid intake (MD = − 0.42; 95% CI − 0.76 to − 0.08; *P* = 0.02; *I*^2^ = 53%) than those in the OG group. While the time to pull gastric tube (MD = − 0.73; 95% CI − 2.17 to 0.72; *P* = 0.32; *I*^2^ = 69%) and drainage tube (MD = − 0.69; 95% CI − 1.46 to 0.08; *P* = 0.08; *I*^2^ = 0%) were not significant different between the two groups.

**Table 3:** Results of the meta-analysis of intraoperative and recovery outcomes

Pooled outcomes	Studies (*n*)	Sample size, *n* (LG:OG)	Effect size with 95% CI	*P* value	Heterogeneity (*I*^2^, %)	Publication bias (Begg’s Test)
Intraoperative outcomes						
Operative time, min (MD)	18	2096 (933:1163)	25.98 (18.42–33.53)	< 0.0001	78	0.820
Estimated blood loss, ml (MD)	18	2096 (933:1163)	− 65.15 (− 91.02 to − 39.27)	< 0.0001	96	0.325
Proximal margin, cm (MD)	5	364 (167:197)	− 0.26 (− 0.60 to 0.08)	0.13	0	–
Distal margin, cm (MD)	5	364 (167:197)	− 0.20 (− 0.65 to 0.25)	0.38	0	–
R1/R2 rate, n (OR)	10	1026 (484:542)	1.47 (0.67 to 3.20)	0.34	0	0.368
Number of retrieved lymph nodes, n (MD)	16	1672 (832:840)	− 0.16 (− 1.43 to 1.10)	0.80	55	0.620
Postoperative recovery outcomes						
Time to first flatus, day (MD)	12	1240 (605:635)	− 0.56 (− 0.77 to − 0.35)	< 0.0001	66	0.062
Time to first liquid intake, day (MD)	10	955 (473:482)	− 0.42 (− 0.76 to − 0.08)	0.02	53	0.371
Time to remove gastric tube, day (MD)	3	452 (138:314)	− 0.73 (− 2.17 to 0.72)	0.32	69	–
Time to remove drainage tube, day (MD)	4	317 (146:171)	− 0.69 (− 1.14 to 0.08)	0.08	0	–
Postoperative hospital stay, day (MD)	18	2096 (933:1163)	− 2.26(− 3.60 to − 0.92)	< 0.0001	97	0.363

#### Postoperative complications

**Fig. 4:**
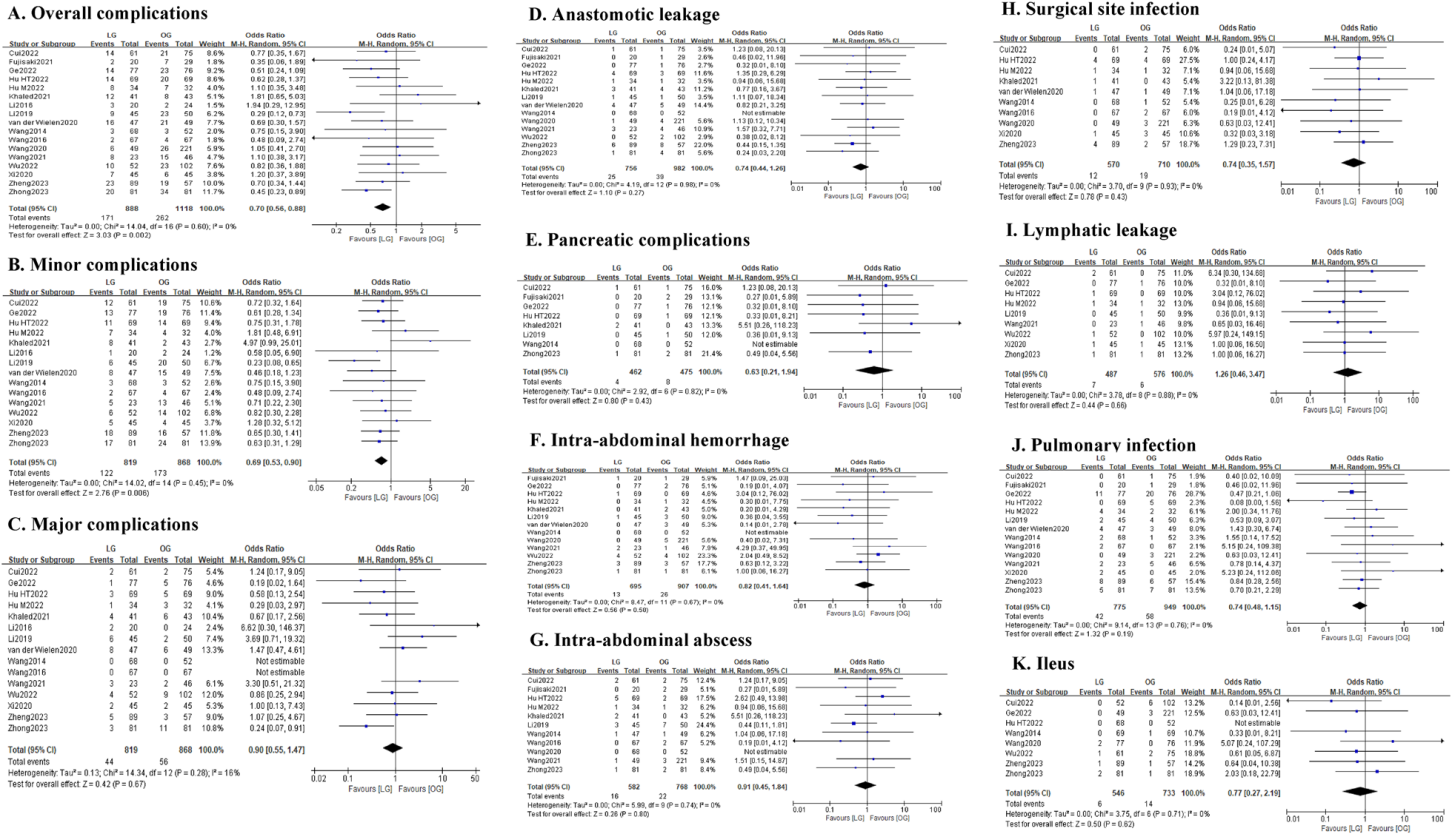
Forest plot assessing postoperative complications between the LG and OG groups. **A** Overall complications; **B** minor complications; **C** major complications; **D** anastomotic leakage; **E** pancreatic complications; **F** intra-abdominal hemorrhage; **G** intra-abdominal abscess; **H** surgical site infection; **I** lymphatic leakage; **J** pulmonary infection; and **K** Ileus

As shown in Fig. 4 and Table 4, a lower overall postoperative complication rate (OR = 0.70; 95% CI 0.56–0.88; *P* = 0.002; *I*^2^ = 0%) and a lower minor complication rate (OR = 0.69; 95% CI 0.53–0.90; *P* = 0.006; *I*^2^ = 0%) were observed in the LG group. While for major complications, there was no significant difference observed between the two groups (OR = 0.90; 95% CI 0.55–1.47; *P* = 0.67; *I*^2^ = 16%). In terms of specific postoperative complications, we found that the incidence of anastomotic leakage (OR = 0.74; 95% CI 0.44–1.26; *P* = 0.27; *I*^2^ = 0%), pancreatic complications (OR = 0.63; 95% CI 0.21–1.94; *P* = 0.43; *I*^2^ = 0%), intra-abdominal abscess (OR = 0.91; 95% CI 0.45–1.84; *P* = 0.80; *I*^2^ = 0%), intra-abdominal bleeding (OR = 0.82; 95% CI 0.41–1.64; *P* = 0.58; *I*^2^ = 0%), lymphatic leakage (OR = 1.26; 95% CI 0.46–3.47; *P* = 0.66; *I*^2^ = 0%), ileus (OR = 0.77; 95% CI 0.27–2.19; *P* = 0.62; *I*^2^ = 0%), surgical site infection (OR = 0.74; 95% CI 0.35–1.57; *P* = 0.43; *I*^2^ = 0%) and pulmonary infection (OR = 0.74; 95% CI 0.48–1.15; *P* = 0.62; *I*^2^ = 0%) were all comparable between the two groups.

**Table 4:** Results of the meta-analysis of postoperative complications

Pooled outcomes	Studies (*n*)	Sample size, *n* (LG: OG)	Effect size with 95% CI	P value	Heterogeneity (*I*^2^, %)	Publication bias (Begg’s Test)
Total complications, *n* (OR)	17	2006 (888:1118)	0.70 (0.56–0.88)	0.002	0	0.064
Minor complications, *n* (OR)	15	1687 (819:868)	0.69 (0.53–0.90)	0.006	0	0.075
Major complications, *n* (OR)	15	1687 (819:868)	0.90 (0.55–1.47)	0.67	16	0.583
Anastomotic leakage, *n* (OR)	14	1738 (756:982)	0.74 (0.44–1.26)	0.27	0	0.502
Pancreatic complications, *n* (OR)	8	937 (462:475)	0.63 (0.21–1.94)	0.43	0	–
Intra-abdominal hemorrhage, *n* (OR)	13	1602 (695:907)	0.82 (0.41–1.64)	0.58	0	0.945
Intra-abdominal abscess, *n* (OR)	11	1350 (582:768)	0.91 (0.45–1.84)	0.80	0	0.858
Surgical site infection, *n* (OR)	10	1280 (570:710)	0.74 (0.35–1.57)	0.43	0	0.474
Lymphatic leakage, *n* (OR)	9	1063 (487:576)	1.26 (0.46–3.47)	0.66	0	–
Pulmonary infection, *n* (OR)	14	1724 (775:949)	0.74 (0.48–1.15)	0.19	0	0.274
Ileus, *n* (OR)	8	1279 (546:733)	0.77 (0.27–2.19)	0.62	0	–

#### Long-term outcomes

**Fig. 5:**
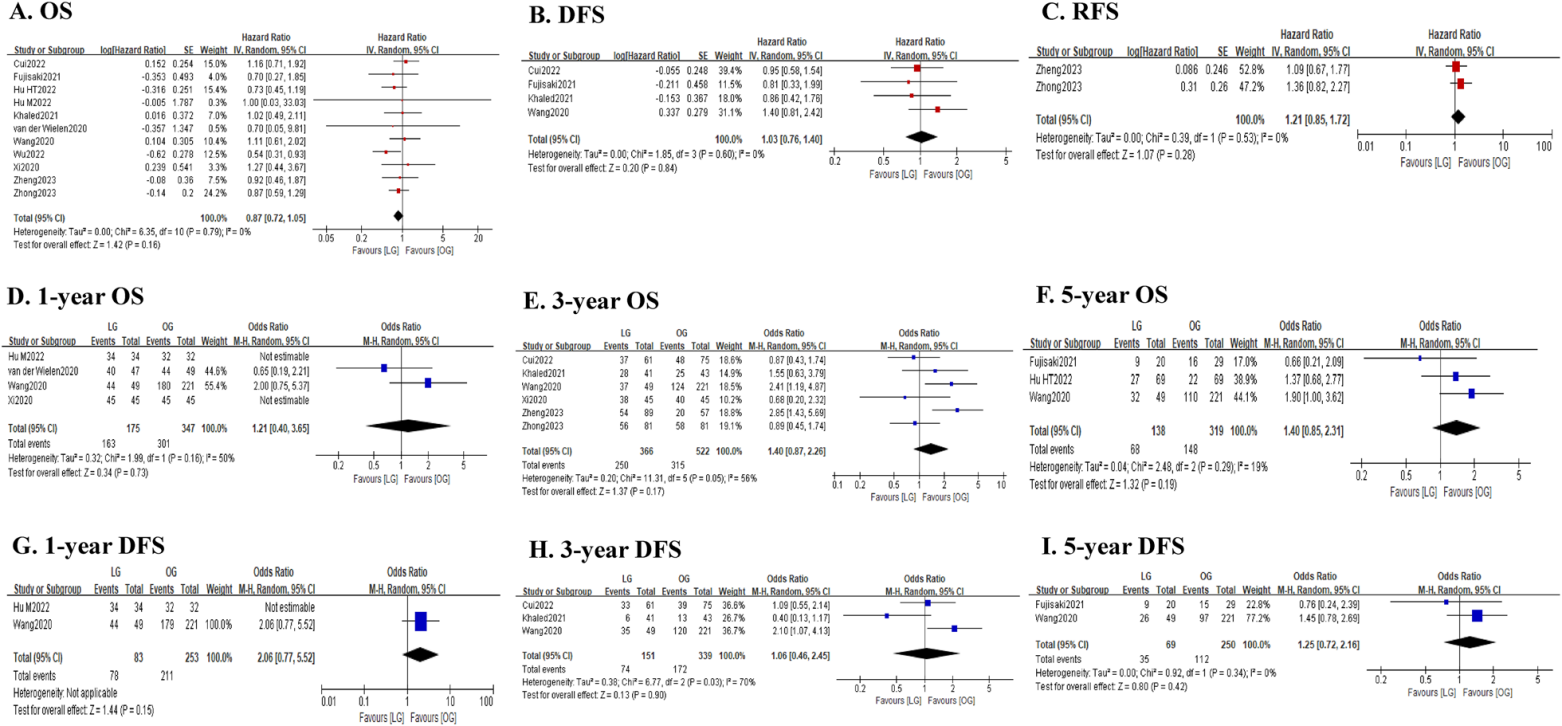
Forest plot assessing long-term survival outcomes between the LG and OG groups. **A** OS; **B** DFS; **C** RFS; **D** 1-year OS rate; **E** 3-year OS rate; **F** 5-year OS rate; **G** 1-year DFS rate; **H** 3-year DFS rate; and **I** 5-year DFS rate

As shown in Fig. 5 and Table 5, the pooled analysis showed that there was no significant difference between the LG and OG groups in terms of OS (HR = 0.87; 95% CI 0.72–1.05; *P* = 0.16; *I*^2^ = 0%). Consistently, the 1-year OS rate (OR = 1.21; 95% CI 0.40–3.65; *P* = 0.73; *I*^2^ = 50%), 3-year OS rate (OR = 1.40; 95% CI 0.87–2.26; *P* = 0.17; *I*^2^ = 56%) and 5-year OS rate (OR = 1.40; 95% CI 0.85–2.31; *P* = 0.19; *I*^2^ = 19%) were also comparable between the two groups. Similarly, the DFS was also comparable between the two groups (HR = 1.03; 95% CI 0.76–1.40; *P* = 0.84; *I*^2^ = 0%). And the 1-year DFS rate (OR = 2.06; 95% CI 0.77–5.52; *P* = 0.15), 3-year DFS rate (OR = 1.06; 95% CI 0.46–2.45; *P* = 0.90; *I*^2^ = 70%) and 5-year DFS rate (OR = 1.25; 95% CI 0.72–2.16; *P* = 0.42; *I*^2^ = 0%) were all comparable between the two groups. In addition, the pooled analysis revealed that the RFS was not significantly different between the two groups (HR = 1.21; 95% CI 0.85–1.62; *P* = 0.28; *I*^2^ = 0%), with comparable 3-year RFS rate (OR = 0.85; 95% CI 0.45–1.62; *P* = 0.62).

**Table 5:** Results of the meta-analysis of long-term survival outcomes

Pooled outcomes	Studies (*n*)	Sample size, *n* (LG:OG)	Effect size with 95% CI	*P* value	Heterogeneity (*I*^2^, %)	Publication bias (Begg’s *P* value)
Overall survival, month (HR)	11	1391 (588:803)	0.87 (0.72–1.05)	0.16	0	1.000
1-year, *n* (OR)	4	522 (175:347)	1.21 (0.40–3.65)	0.73	50	–
3-year, *n* (OR)	6	888 (366:522)	1.40 (0.87–2.26)	0.17	56	–
5-year, *n* (OR)	3	457 (138:319)	1.40 (0.85–2.31)	0.19	19	–
Disease-free survival, month (HR)	4	539 (171:368)	1.03 (0.76–1.40)	0.84	0	–
1-year, *n* (OR)	2	336 (83:253)	2.06 (0.77–5.52)	0.15	–	–
3-year, *n* (OR)	3	490 (151:339)	1.06 (0.46–2.45)	0.90	70	–
5-year, *n* (OR)	2	319 (69:250)	1.25 (0.72–2.16)	0.42	0	–
Recurrence-free survival, month (HR)	2	308 (170:138)	1.21 (0.85–1.72)	0.28	0	–
3-year, *n* (OR)	1	162 (81:81)	0.85 (0.45–1.62)	0.62	–	–

#### Meta-regression and subgroup analyses

Since high heterogeneity was observed in the meta-analyses, especially in operative time, estimated blood loss, retrieved lymph nodes, time to first flatus, time to first liquid intake and postoperative hospital stay, multivariate meta-regression analyses based on publication year (≥ 2020 vs. < 2020), county (China vs. Others), study design (RCT/PSM cohort vs. Others), sample size (> 100 vs. ≤ 100), the extent of gastrectomy (total gastrectomy vs. others) and baseline features (matched vs. unmatched) were performed. As shown in Additional file 1: Table S1, sample size was the major source of heterogeneity of retrieved lymph nodes (*P* = 0.022), while study design (*P* = 0.035) and baseline features (*P* = 0.009) were the major source of heterogeneity of postoperative hospital stay.

Furthermore, subgroup analyses stratified by those covariates were performed to investigate the discrepant treatment effect of different subsets. As shown in Additional file 1: Figs. S1–6, the findings of all subgroup analyses were consistent with the results of subgroup analyses and suggested that these perioperative outcomes in the LG group were not inferior to those in the OG group, except that the LG group showed a lower rate of retrieved lymph nodes in the subset with a sample size of no more than 100 (MD = − 1.94; 95% CI − 3.32 to − 0.56; *P* = 0.006; *I*^2^ = 14%).

### Publication bias

For pooled outcomes with at least ten studies included, the Begg’s funnel plot was applied to assess the potential publication bias. As shown in Tables 3, 4, 5 and Additional file 1: Fig. S7, all of the pooled outcomes showed no significant risk of publication bias, with all p-values greater than 0.05.

## Discussion

In clinical practice, laparoscopic gastrectomy has been widely accepted as an alternative to open gastrectomy for AGC in both Eastern and Western countries due to its relatively better short-term outcomes without compromising long-term outcomes. [44–46]. Nowadays, the application of NAT to AGC patients has rapidly increased owing to its potential oncological benefits [5, 28, 31]. Nevertheless, before applying LG as the standard treatment option for AGC patients receiving NAT, much more evidence is required to confirm the true benefits of LG over OG, thereby aiding surgical decision-making.

In the present meta-analysis, we enrolled 18 studies with 2096 patients and demonstrated that LG offers better perioperative outcomes and comparable survival results compared to OG. In detail, the present study highlighted less estimated blood loss, faster time to liquid intake, shorter length of hospital stay, fewer minor complication rate, but longer operative time in the LG group, except for a reduced overall complication rate and faster time to flatus. Nonetheless, the previous meta-analysis [18] failed to detect these differences due to the limited number of studies included. Also, benefiting from a larger sample size, informative meta-regression and subgroup analyses can be further performed to investigate sources of heterogeneity and robustness of these pooled results with significant heterogeneities. As shown in Additional file 1: Figs. S1–S6, nearly all of those subset analyses revealed that these perioperative outcomes in the LG group were not inferior to those in the OG group. In addition, Begg’s tests showed no significant risk of bias in those pooled results, which further convincing us of the feasibility of LG in AGC patients following NAT.

Surveying the surgical parameters, the estimated blood loss in the LG group was significantly less than that in the OG group. This result may be related to the fine dissection and meticulous hemostasis of edematous and fibrotic tissue under a magnified surgical view by advanced laparoscopic instruments [26]. In addition, the LG group showed a faster recovery of gastrointestinal function, as indicated by the earlier onset of flatus and oral intake. Time to pull gastric tube and drainage tube, however, was similar between the two groups. Moreover, LG was also associated with a significant reduction in hospital stay, which may be a combined result of less blood loss, faster recovery of bowel function and lower postoperative complication rate. On the other hand, a longer operative time was observed in the LG group. Numerous studies have shown that the prolonged operative time is closely associated with the technical complexity as well as a relatively long learning curve of laparoscopic surgery [47], which may be particularly true in the context of NAT. With the popularization of laparoscopic technique and the improvement of surgical proficiency of surgeons, the operative time is expected to be shortened in future clinical practice.

Regarding postoperative morbidity, LG was related to a lower overall complication rate and a lower minor complication rate. These results may be related to the inherent benefit of laparoscopy in terms of better exposure and visual magnification, which allows delicate manipulation of the organs, vessels and nerves during LG [48]. In addition, the use of sophisticated equipment such as Harmonic Scalpel and Ligasure during LG may facilitate decreasing the surgery damage to normal tissues, therefore reducing morbidity [49]. Nevertheless, it is not surprising that in our pooled result, the minimally invasive nature of LG had less impact on reducing major complications, because accumulating reports have suggested that the occurrence of major complications may be mainly related to the proficiency of the surgeons as well as the surgical devices, rather than the surgical approach [48, 50]. In terms of specific complications, although there were no significant differences between the two groups found in our study, lower odds ratios for most complications were observed in the LG group, such as pulmonary infection. Certainly, part of the reason for this evidence can be explained by the minimally invasive nature of laparoscopic approach, which allows minor surgical incision length and less immune-suppression [48]. Besides, the minor tension sutures can reduce postoperative pain, thereby improving the patient’s respiratory dynamics and leading to fewer pulmonary infection [51].

With respect to the evaluation of oncological adequacy, our results demonstrated that LG was equal to OG in the proximal margin, distal margin, R1/R2 resection rate and number of harvested lymph nodes. In particular, retrieving enough lymph nodes for pathological examination and achieving identical extent of lymphadenectomy to OG have been regarded as the most essential index for assessing the feasibility of LG in gastric cancer patients [52–54]. The 8th AJCC guideline recommends that at least 16 lymph nodes are required for GC patients to ensure accurate N staging, regardless of receiving NAT or not [11]. In addition, a recent study involving 4337 cases suggested that the retrieval of at least 23 lymph nodes could provide a better survival for patients receiving NAT [55]. In our study, the mean number of lymph nodes retrieved in the LG and OG groups was 31.84 and 31.87, indicating that LG is as oncologically adequate as OG in patients undergoing NAT. Consistently, the OS, DFS and RFS rates between the two approaches were unsurprisingly comparable. Therefore, once the basic principles of negative margins and adequate lymphadenectomy have been secured, the survival results are largely determined by the biological characteristics of the tumor itself rather than by the surgical approach [47].

This meta-analysis has several limitations. First, among included studies, there were only 2 RCTs and most of them were retrospective in nature, which may increase the risk of selective bias. Second, the quality of the included studies varied. Even though those pooled results were of low heterogeneities or remained consistent in the subgroup of high-quality studies (RCTs and propensity-score matched studies), this may have some effect on the strength of evidence of our study. Third, the preoperative treatment regimens varied a lot among the included studies. This heterogeneity may have an impact on the perioperative and survival outcome analyses.

## Conclusions

This meta-analysis suggests that LG is a safe and feasible technique for AGC patients who received NAT in terms of superior short-term and comparable long-term results. Nevertheless, high-quality multicenter RCTs are warranted to validate our findings.

## Supplementary Information

**Additional file 1. **Additional Tables and Figures.

## Data Availability

The datasets used and/or analyzed during the current study are available from the corresponding author on reasonable request.

## Abbreviations

GC: Gastric cancer
NAT: Neoadjuvant treatment
AGC: Advanced gastric cancer
LG: Laparoscopic gastrectomy
OG: Open gastrectomy
BMI: Body mass index
ASA: American Society of Anesthesiologists classification
OS: Overall survival
DFS: Disease-free survival
RFS: Recurrence-free survival
CD: Clavien–Dindo
RCT: Randomized controlled trial
RoB 2.0: Risk-of-Bias 2.0
ROBINS-I: Risk of Bias in Non-Randomized Studies-of Interventions
OR: Odds ratio
MD: Mean difference
HR: Hazard ratio
CI: Confidence interval
SD: Standard deviation

## References

[1] Sung H, Ferlay J, Siegel RL (2021). Global cancer statistics 2020: GLOBOCAN estimates of incidence and mortality worldwide for 36 cancers in 185 countries. CA Cancer J Clin.

[2] Pang H, Zhang W, Liang X (2021). Prognostic Score System using preoperative inflammatory, nutritional and tumor markers to predict prognosis for gastric cancer: a two-center cohort study. Adv Ther.

[3] Marano L, D’Ignazio A, Cammillini F (2019). Comparison between 7th and 8th edition of AJCC TNM staging system for gastric cancer: old problems and new perspectives. Transl Gastroenterol Hepatol.

[4] Li CC, Yeh YS, Chen YC (2022). Surgical efficacy and safety of patients with locally advanced gastric cancer following neoadjuvant concurrent chemoradiotherapy and chemotherapy. J Oncol.

[5] Cunningham D, Allum WH, Stenning SP (2006). Perioperative chemotherapy versus surgery alone for resectable gastroesophageal cancer. N Engl J Med.

[6] Al-Batran SE, Homann N, Pauligk C (2019). Perioperative chemotherapy with fluorouracil plus leucovorin, oxaliplatin, and docetaxel versus fluorouracil or capecitabine plus cisplatin and epirubicin for locally advanced, resectable gastric or gastro-oesophageal junction adenocarcinoma (FLOT4): a randomised, phase 2/3 trial. Lancet.

[7] Wang X, Li S, Sun Y (2021). The protocol of a prospective, multicenter, randomized, controlled phase III study evaluating different cycles of oxaliplatin combined with S-1 (SOX) as neoadjuvant chemotherapy for patients with locally advanced gastric cancer: RESONANCE-II trial. BMC Cancer.

[8] Zhang X, Liang H, Li Z (2021). Perioperative or postoperative adjuvant oxaliplatin with S-1 versus adjuvant oxaliplatin with capecitabine in patients with locally advanced gastric or gastro-oesophageal junction adenocarcinoma undergoing D2 gastrectomy (RESOLVE): an open-label, superiority and non-inferiority, phase 3 randomised controlled trial. Lancet Oncol.

[9] Miao ZF, Liu XY, Wang ZN (2018). Effect of neoadjuvant chemotherapy in patients with gastric cancer: a PRISMA-compliant systematic review and meta-analysis. BMC Cancer.

[10] Fong C, Johnston E, Starling N (2022). Neoadjuvant and adjuvant therapy approaches to gastric cancer. Curr Treat Options Oncol.

[11] Ajani JA, D’Amico TA, Bentrem DJ (2022). Gastric cancer, Version 2.2022, NCCN clinical practice guidelines in oncology. J Natl Compr Canc Netw.

[12] Kitano S, Iso Y, Moriyama M, Sugimachi K (1994). Laparoscopy-assisted Billroth I gastrectomy. Surg Laparosc Endosc.

[13] Inaki N, Etoh T, Ohyama T (2015). A multi-institutional, prospective, Phase II feasibility study of laparoscopy-assisted distal gastrectomy with D2 lymph node dissection for locally advanced gastric cancer (JLSSG0901). World J Surg.

[14] Hyung WJ, Yang HK, Park YK (2020). Long-term outcomes of laparoscopic distal gastrectomy for locally advanced gastric cancer: the KLASS-02-RCT randomized clinical trial. J Clin Oncol.

[15] Yu J, Huang C, Sun Y (2019). Effect of Laparoscopic vs open distal gastrectomy on 3-year disease-free survival in patients with locally advanced gastric cancer: the CLASS-01 randomized clinical trial. JAMA.

[16] Coccolini F, Nardi M, Montori G (2018). Neoadjuvant chemotherapy in advanced gastric and esophago-gastric cancer. Meta-analysis of randomized trials. Int J Surg.

[17] Reddavid R, Sofia S, Chiaro P (2018). Neoadjuvant chemotherapy for gastric cancer. Is it a must or a fake?. World J Gastroenterol.

[18] Liao XL, Liang XW, Pang HY (2021). Safety and efficacy of laparoscopic versus open gastrectomy in patients with advanced gastric cancer following neoadjuvant chemotherapy: a meta-analysis. Front Oncol.

[19] Amir-Behghadami M, Janati A (2020). Population, intervention, comparison, outcomes and study (PICOS) design as a framework to formulate eligibility criteria in systematic reviews. Emerg Med J.

[20] Clavien PA, Barkun J, de Oliveira ML (2009). The Clavien-Dindo classification of surgical complications: five-year experience. Ann Surg.

[21] Sterne JAC, Savović J, Page MJ (2019). RoB 2: a revised tool for assessing risk of bias in randomised trials. BMJ.

[22] Sterne JA, Hernán MA, Reeves BC (2016). ROBINS-I: a tool for assessing risk of bias in non-randomised studies of interventions. BMJ.

[23] McGrath S, Zhao X, Steele R (2020). Estimating the sample mean and standard deviation from commonly reported quantiles in meta-analysis. Stat Methods Med Res.

[24] Tierney JF, Stewart LA, Ghersi D (2007). Practical methods for incorporating summary time-to-event data into meta-analysis. Trials.

[25] Higgins JP, Thompson SG, Deeks JJ, Altman DG (2003). Measuring inconsistency in meta-analyses. BMJ.

[26] Li Z, Shan F, Ying X (2019). Assessment of laparoscopic distal gastrectomy after neoadjuvant chemotherapy for locally advanced gastric cancer: a randomized clinical trial. JAMA Surg.

[27] van der Wielen N, Straatman J, Daams F (2021). Open versus minimally invasive total gastrectomy after neoadjuvant chemotherapy: results of a European randomized trial. Gastric Cancer.

[28] Cui H, Zhang KC, Cao B (2022). Short and long-term outcomes between laparoscopic and open total gastrectomy for advanced gastric cancer after neoadjuvant chemotherapy. World J Gastrointest Surg.

[29] Fujisaki M, Mitsumori N, Shinohara T (2021). Short- and long-term outcomes of laparoscopic versus open gastrectomy for locally advanced gastric cancer following neoadjuvant chemotherapy. Surg Endosc.

[30] Ge R, Liu K, Zhang W (2022). The safety and feasibility of laparoscopic gastrectomy after neoadjuvant chemotherapy for locally advanced gastric cancer. J Oncol.

[31] Hu HT, Ma FH, Xiong JP (2022). Laparoscopic vs open total gastrectomy for advanced gastric cancer following neoadjuvant therapy: a propensity score matching analysis. World J Gastrointest Surg.

[32] Hu MX, Tian; Li, Tengteng; Wang, Kai, Fu, Haixiao; Zhang, Xuan; Fu, Wei. Comparison of safety and short-term efficacy between laparoscopy and laparotomy for locally advanced proximal gastric cancer after neoadjuvant chemotherapy. 2022; 49: D.

[33] Jingxia Shen HK, Xuhui Liu, Liyun Ling, Bin Wei, Huisheng Wang. Clinical efficacy of neoadjuvant chemotherapy combined with laparoscopy in the treatment of proximal advanced gastric cancer. 2020.

[34] Khaled I, Priego P, Soliman H (2021). Oncological outcomes of laparoscopic versus open gastrectomy after neoadjuvant chemotherapy for locally advanced gastric cancer: a retrospective multicenter study. World J Surg Oncol.

[35] Li Z, Shan F, Wang Y (2016). Laparoscopic versus open distal gastrectomy for locally advanced gastric cancer after neoadjuvant chemotherapy: safety and short-term oncologic results. Surg Endosc.

[36] Wang JH, Xiaopeng; Su, Lin; Li, Hongtao; Yu, Jianping; Li, Sandang; Liu, Hongbin. Comparison of the recent treatment effect between laparoscopic and open surgery following neoadjuvant chemortherapy for advanced gastric cancer. 2014; 22.

[37] Wang N, Zhou A, Jin J (2020). Open vs. laparoscopic surgery for locally advanced gastric cancer after neoadjuvant therapy: short-term and long-term survival outcomes. Oncol Lett.

[38] Wang Y. Clinical study of platinum-based neoadjuvant chemotherapy combined with laparoscopic resection in the treatment of primary gastric cancer. 2016.

[39] Wang Y, Lei X, Liu Z (2021). Short-term outcomes of laparoscopic versus open total gastrectomy after neoadjuvant chemotherapy: a cohort study using the propensity score matching method. J Gastrointest Oncol.

[40] Wu S. Safety and efficacy of laparoscopy and laparotomy after neoadjuvant chemotherapy for locally advanced gastric cancer. Academic Dissertation 2022.

[41] Xi HQ, Zhang KC, Li JY (2020). Comparison of perioperative and survival outcomes of laparoscopic versus open gastrectomy after preoperative chemotherapy: a propensity score-matched analysis. Indian J Surg.

[42] Zheng HL, Shen LL, Xu BB (2023). Oncological outcomes of laparoscopic versus open radical total gastrectomy for upper-middle gastric cancer after neoadjuvant chemotherapy: a study of real-world data. Surg Endosc.

[43] Zhong H, Liu X, Tian Y (2023). Comparison of short- and long-term outcomes between laparoscopic and open gastrectomy for locally advanced gastric cancer following neoadjuvant chemotherapy: a propensity score matching analysis. Surg Endosc.

[44] Pang HY, Zhao LY, Zhang ZQ (2021). Comparisons of perioperative and survival outcomes of laparoscopic versus open gastrectomy for serosa-positive (pT4a) gastric cancer patients: a propensity score matched analysis. Langenbecks Arch Surg.

[45] van der Veen A, Brenkman HJF, Seesing MFJ (2021). Laparoscopic versus open gastrectomy for gastric cancer (LOGICA): a multicenter randomized clinical trial. J Clin Oncol.

[46] Huang C, Liu H, Hu Y (2022). Laparoscopic vs open distal gastrectomy for locally advanced gastric cancer: five-year outcomes from the CLASS-01 randomized clinical trial. JAMA Surg.

[47] Chen X, Feng X, Wang M, Yao X (2020). Laparoscopic versus open distal gastrectomy for advanced gastric cancer: a meta-analysis of randomized controlled trials and high-quality nonrandomized comparative studies. Eur J Surg Oncol.

[48] Caruso S, Giudicissi R, Mariatti M (2022). Laparoscopic vs. open gastrectomy for locally advanced gastric cancer: a propensity score-matched retrospective case-control study. Curr Oncol.

[49] Zeng YK, Yang ZL, Peng JS (2012). Laparoscopy-assisted versus open distal gastrectomy for early gastric cancer: evidence from randomized and nonrandomized clinical trials. Ann Surg.

[50] Chen K, Xu XW, Zhang RC (2013). Systematic review and meta-analysis of laparoscopy-assisted and open total gastrectomy for gastric cancer. World J Gastroenterol.

[51] Best LM, Mughal M, Gurusamy KS (2016). Laparoscopic versus open gastrectomy for gastric cancer. Cochrane Database Syst Rev.

[52] Sato H, Shimada M, Kurita N (2012). Comparison of long-term prognosis of laparoscopy-assisted gastrectomy and conventional open gastrectomy with special reference to D2 lymph node dissection. Surg Endosc.

[53] Park YK, Yoon HM, Kim YW (2018). Laparoscopy-assisted versus Open D2 distal gastrectomy for advanced gastric cancer: results from a randomized phase II multicenter clinical trial (COACT 1001). Ann Surg.

[54] Viñuela EF, Gonen M, Brennan MF (2012). Laparoscopic versus open distal gastrectomy for gastric cancer: a meta-analysis of randomized controlled trials and high-quality nonrandomized studies. Ann Surg.

[55] Shannon AB, Straker RJ, Keele L (2022). Lymph node evaluation after neoadjuvant chemotherapy for patients with gastric cancer. Ann Surg Oncol.

