# Supplementary material for: Comparisons of perioperative and long-term outcomes of laparoscopic versus open gastrectomy for advanced gastric cancer after neoadjuvant therapy: an updated pooled analysis of eighteen studies
Source: Eur J Med Res. 2023 Jul 5;28:224. doi: 10.1186/s40001-023-01197-1 (PMC10320971; doi:10.1186/s40001-023-01197-1)
Supplement: Supplementary file 1 — Additional file 1. Additional Tables and Figures. [file 40001_2023_1197_MOESM1_ESM.docx]

Table S1. Meta-regression of pooled outcomes.

|  | **Operative time** | |  | **Estimated blood loss** | |  | **Number of retrieved lymph nodes** | |  | **Time to first flatus** | |  | **Time to first liquid intake** | |  | **Postoperative hospital stay** | |
| --- | --- | --- | --- | --- | --- | --- | --- | --- | --- | --- | --- | --- | --- | --- | --- | --- | --- |
|  | **P value** | **95%CI** |  | **P value** | **95%CI** |  | **P value** | **95%CI** |  | **P value** | **95%CI** |  | **P value** | **95%CI** |  | **P value** | **95%CI** |
| **Publication year**  (≥2020 vs. <2020) | 0.872 | -31.32-36.42 |  | 0.335 | -208.07-77.37 |  | 0.688 | -2.76-4.00 |  | 0.421 | -1.62-0.83 |  | 0.629 | -2.47-1.76 |  | 0.161 | -0.68-3.67 |
| **Country**  (China vs. others) | 0.905 | -46.97-42.02 |  | 0.402 | -252.61-109.37 |  | 0.617 | -7.35-4.61 |  | 0.319 | -0.75-1.79 |  | 0.607 | -1.92-2.76 |  | 0.816 | -2.90-2.33 |
| **Study design**  (RCT/ PSM vs. others) | 0.523 | -37.83-20.39 |  | 0.880 | -114.79-132.08 |  | 0.261 | -1.32-4.29 |  | 0.172 | -1.37-0.34 |  | 0.458 | -2.80-1.62 |  | **0.035** | **-4.12 to -0.18** |
| **Sample size**  (>100 vs. ≤100) | 0.199 | -48.45-11.32 |  | 0.972 | -127.26-123.12 |  | **0.022** | **0.61-6.12** |  | 0.911 | -0.83-0.90 |  | 0.197 | -0.79-2.50 |  | 0.391 | -2.85-1.20 |
| **Gastrectomy**  (TG vs. others) | 0.269 | -47.69-14.71 |  | 0.088 | -19.23-238.47 |  | 0.462 | -2.27-4.60 |  | 0.961 | -1.12-1.08 |  | 0.799 | -1.59-1.90 |  | 0.537 | -1.43-2.60 |
| **Baseline features***  (Matched vs. unmatched) | 0.964 | -36.35-77.56 |  | 0.248 | -68.65-239.54 |  | 0.695 | -4.11-5.91 |  | 0.878 | -1.50-1.33 |  | 0.485 | -3.57-5.95 |  | **0.009** | **1.03-5.85** |

*Baseline features include age, sex, BMI, ASA, tumor size, gastrectomy extent.


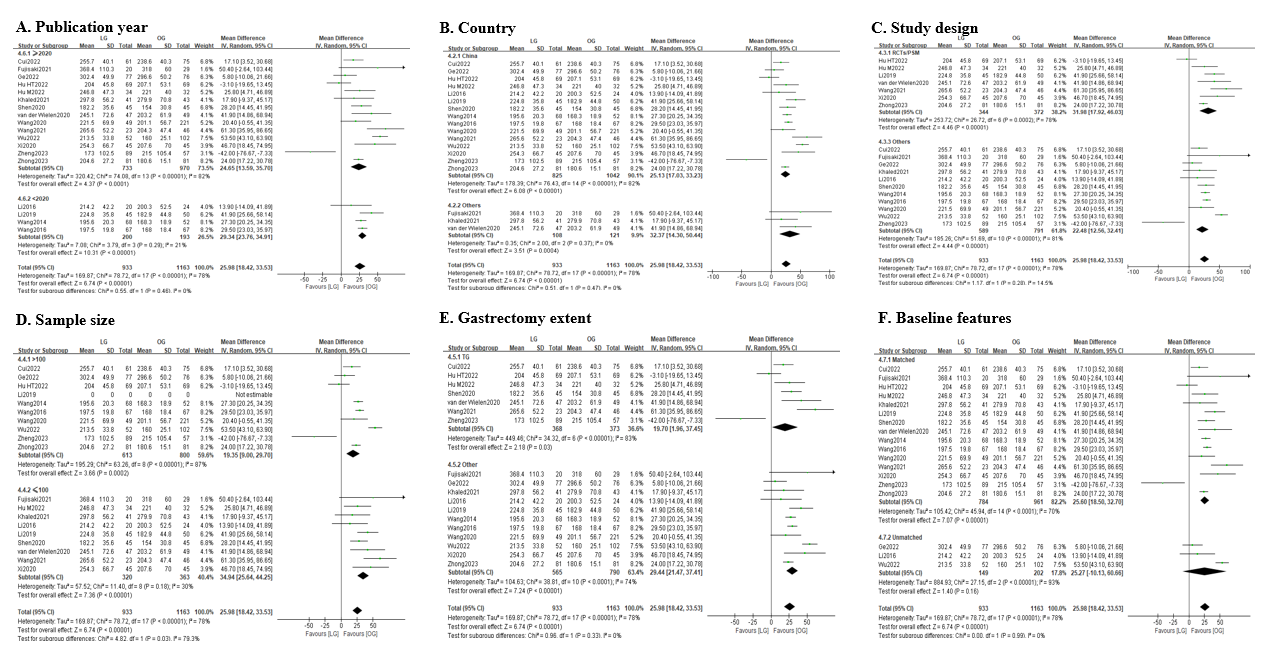


Figure S1. Subgroup analysis of operative time based on A: publication year (≥2020 vs. <2020), B: Country (China vs. Others), C: study design (RCT/PSM cohort vs. Others), D. sample size (>100 vs. ≤100), E: gastrectomy extent (TG vs. Others) and F: baseline features (matched vs. unmatched).


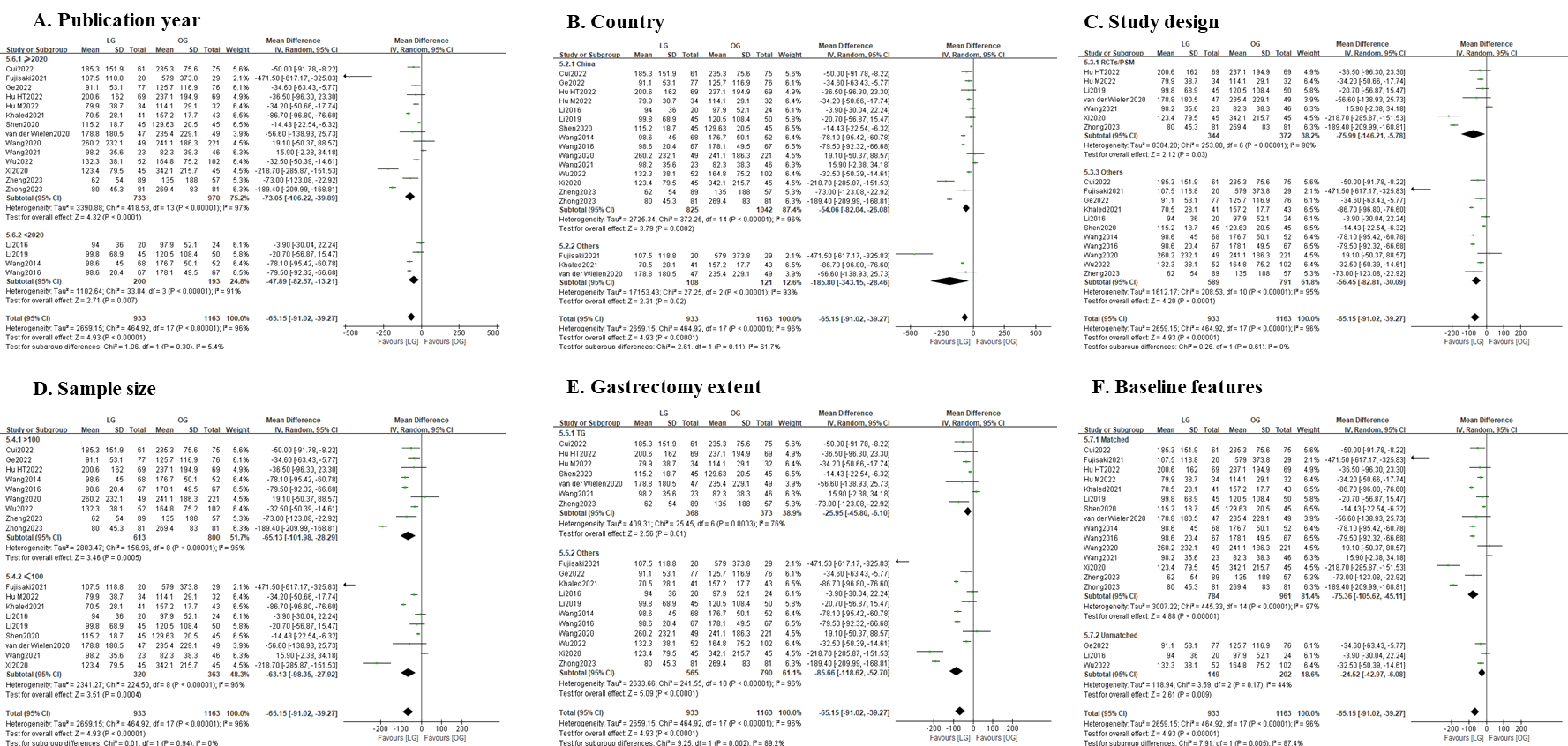


Figure S2. Subgroup analysis of estimated blood loss based on A: publication year (≥2020 vs. <2020), B: Country (China vs. Others), C: study design (RCT/PSM cohort vs. Others), D. sample size (>100 vs. ≤100), E: gastrectomy extent (TG vs. Others) and F: baseline features (matched vs. unmatched).


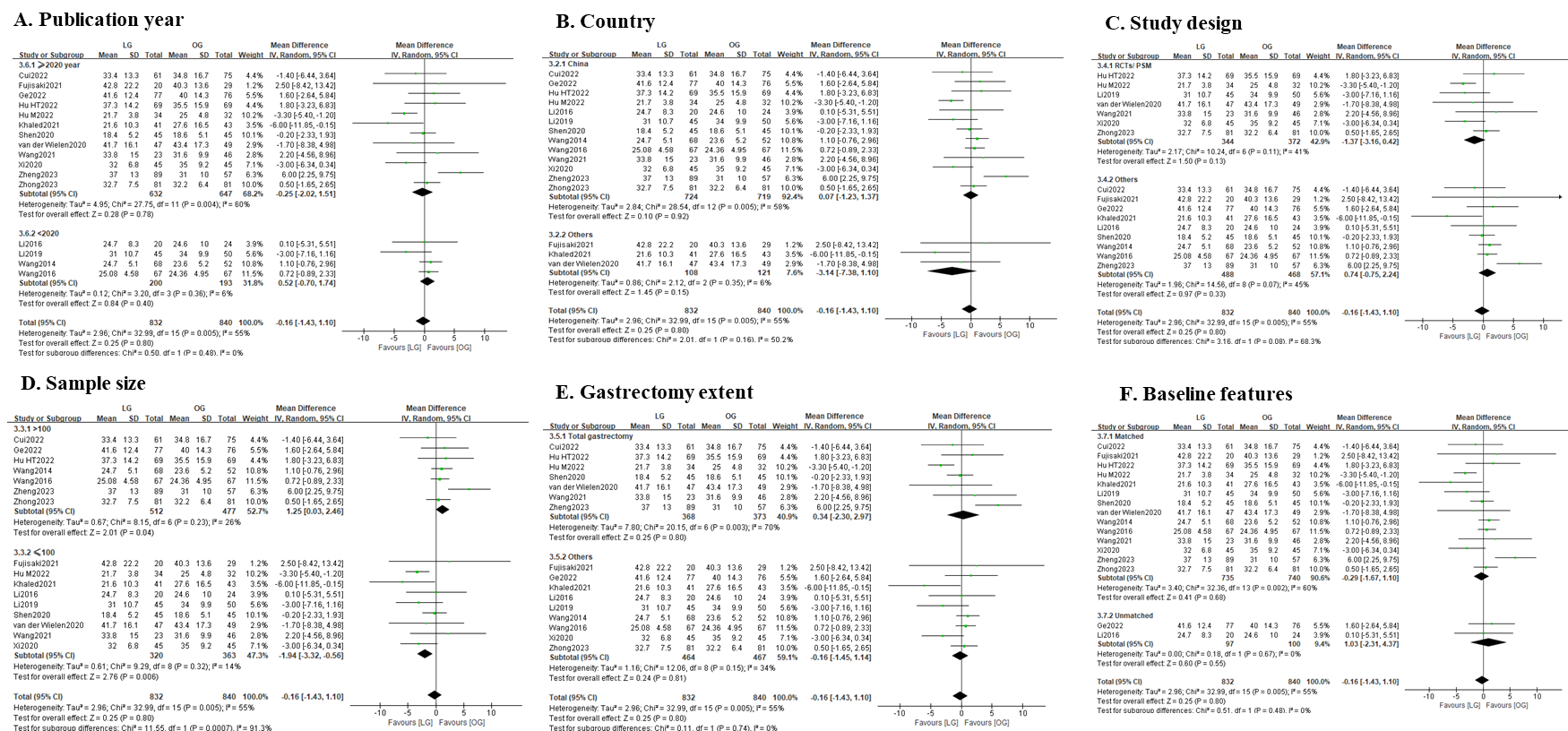


Figure S3. Subgroup analysis of retrieved lymph nodes based on A: publication year (≥2020 vs. <2020), B: Country (China vs. Others), C: study design (RCT/PSM cohort vs. Others), D. sample size (>100 vs. ≤100), E: gastrectomy extent (TG vs. Others) and F: baseline features (matched vs. unmatched).


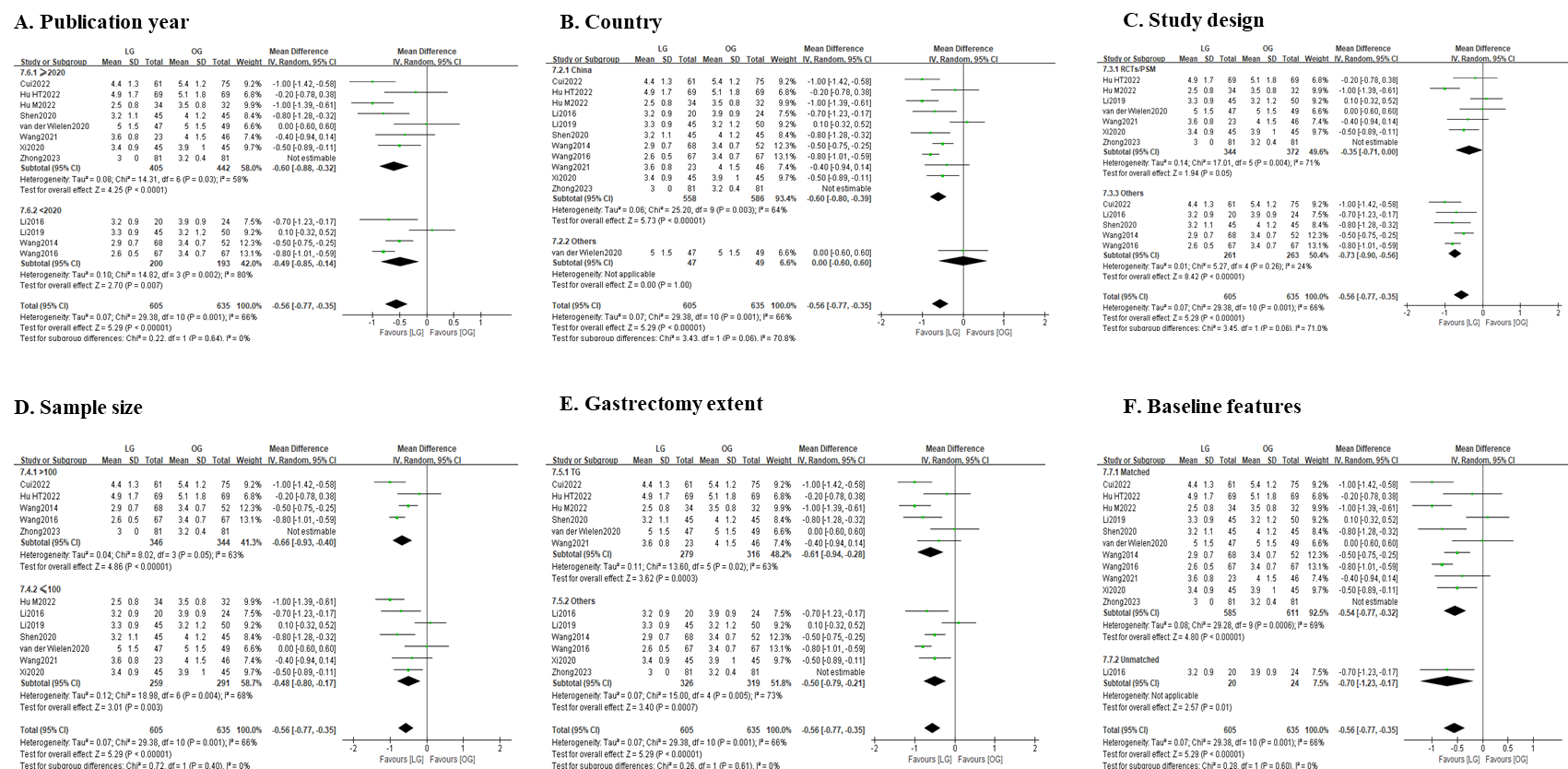


Figure S4. Subgroup analysis of time to first flatus based on A: publication year (≥2020 vs. <2020), B: Country (China vs. Others), C: study design (RCT/PSM cohort vs. Others), D. sample size (>100 vs. ≤100), E: gastrectomy extent (TG vs. Others) and F: baseline features (matched vs. unmatched).


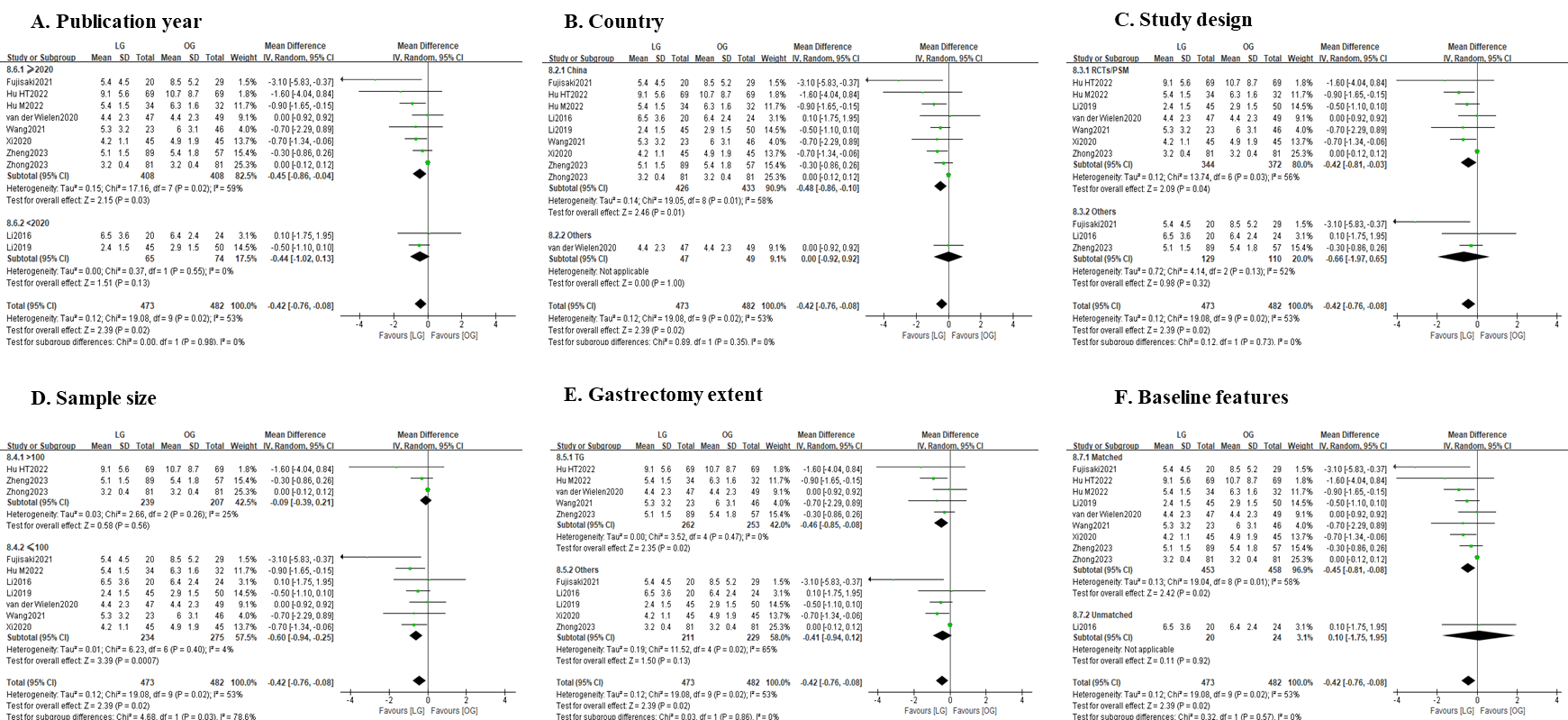


Figure S5. Subgroup analysis of time to first liquid intake based on A: publication year (≥2020 vs. <2020), B: Country (China vs. Others), C: study design (RCT/PSM cohort vs. Others), D. sample size (>100 vs. ≤100), E: gastrectomy extent (TG vs. Others) and F: baseline features (matched vs. unmatched).


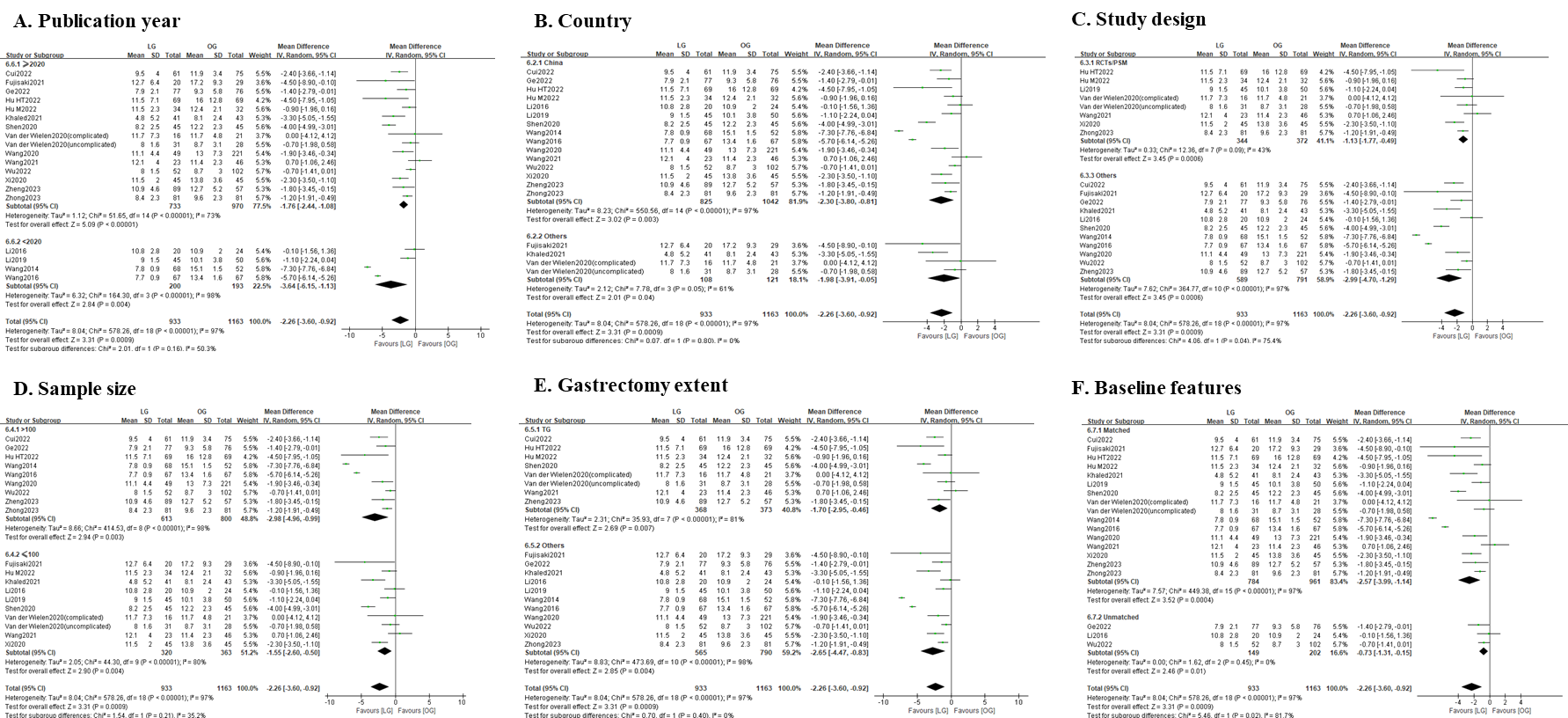


Figure S6. Subgroup analysis of postoperative hospital stay based on A: publication year (≥2020 vs. <2020), B: Country (China vs. Others), C: study design (RCT/PSM cohort vs. Others), D. sample size (>100 vs. ≤100), E: gastrectomy extent (TG vs. Others) and F: baseline features (matched vs. unmatched).


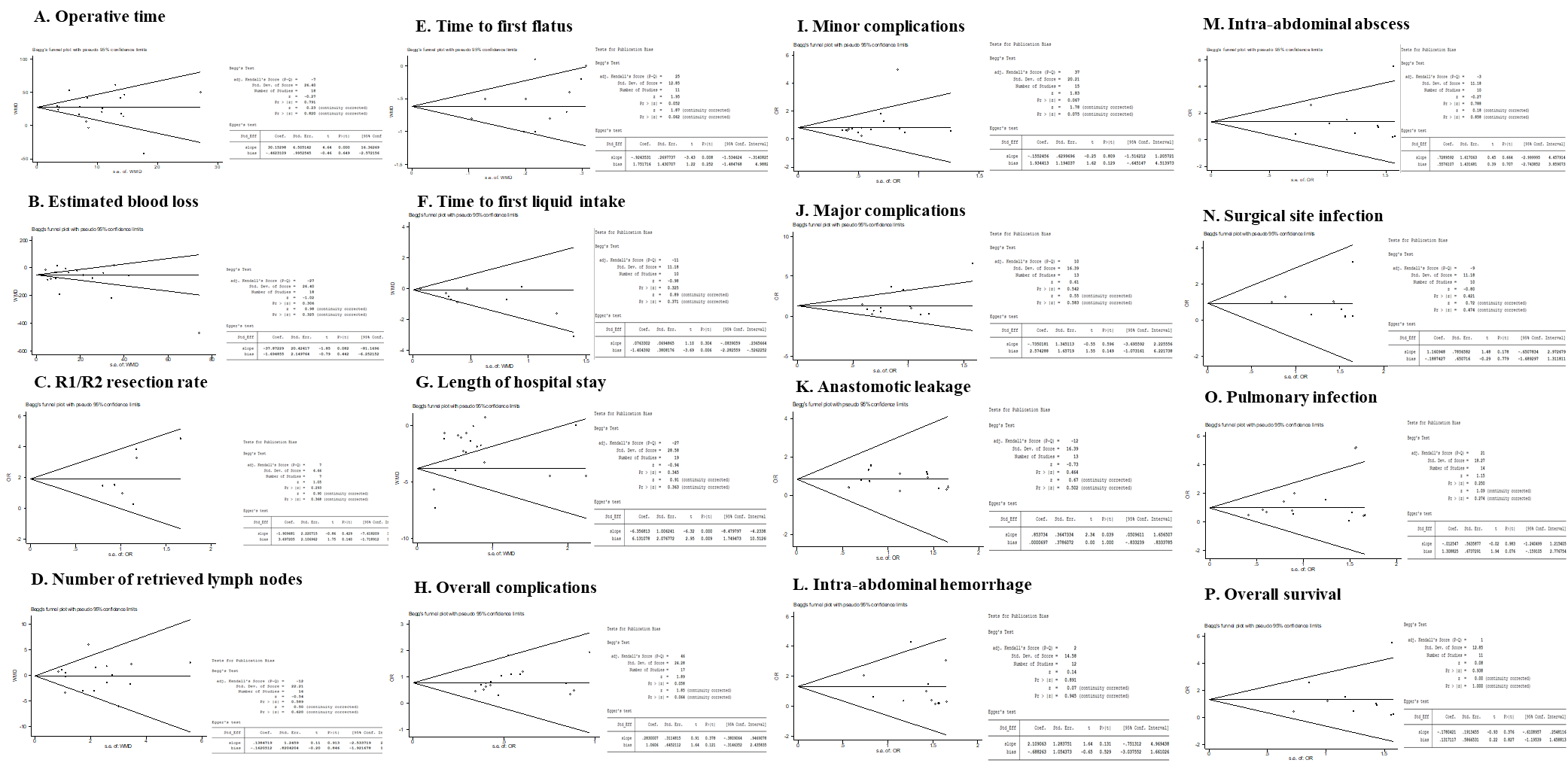


Figure S7. Begg’s funnel plot of perioperative outcomes and long-term survival outcomes. All p values>0.05.
